# A dual-clustering framework for association screening with whole genome sequencing data and longitudinal traits

**DOI:** 10.1186/1753-6561-8-S1-S47

**Published:** 2014-06-17

**Authors:** Ying Liu, ChienHsun Huang, Inchi Hu, Shaw-Hwa Lo, Tian Zheng

**Affiliations:** 1 Department of Statistics, Columbia University, 1255 Amsterdam Avenue, New York, NY 10027, USA; 2 ISOM, Hong Kong University of Science and Technology, Kowloon, Hong Kong

## Abstract

Current sequencing technology enables generation of whole genome sequencing data sets that contain a high density of rare variants, each of which is carried by, at most, 5% of the sampled subjects. Such variants are involved in the etiology of most common diseases in humans. These diseases can be studied by relevant longitudinal phenotype traits. Tests for association between such genotype information and longitudinal traits allow the study of the function of rare variants in complex human disorders. In this paper, we propose an association-screening framework that highlights the genotypic differences observed on rare variants and the longitudinal nature of phenotypes. In particular, both variants within a gene and longitudinal phenotypes are used to create partitions of subjects. Association between the 2 sets of constructed partitions is then evaluated. We apply the proposed strategy to the simulated data from the Genetic Analysis Workshop 18 and compare the obtained results with those from sequence kernel association test using the receiver operating characteristic curves.

## Background

Rare variants have been speculated to be involved in the etiology of complex human diseases [1]. Such diseases usually progress over time so that measures of relevant traits at different time points can provide information on the disease development process. For example, the Type 2 Diabetes Genetic Exploration by Next-generation sequencing in Ethnic Samples (T2D-GENES) Project 2 aims to identify rare variants influencing susceptibility to type 2 diabetes using information from whole genome sequencing (WGS) and measurements of related traits (such as blood pressure) at up to 4 time points. Such WGS genotype and longitudinal phenotype data present new challenges to commonly used statistical methods for association testing in genome-wide studies.

Many genetic variants are rare variants (here we are referring to rare variants with minor allele frequencies [MAFs] <5%). Because of their low MAFs, traditional association methods may suffer from low power. A natural idea for improving power is grouping or collapsing together certain variants. Such collapsing methods are based on the assumption that rare variants in a group (eg, gene or pathway) may function in combination [2]. For example, the sequence kernel association test (SKAT) [3] assigns different weights to variants in a region and incorporates them into a kernel matrix. We have proposed an inverse-probability weighted clustering approach [4], a gene-based method where inverse-probability weighting is used to overweigh genotypic differences observed on rare variants. The above methods can deal with both continuous and dichotomous traits and have obtained insightful results in different studies. However, leveraging them in an effort to efficiently address longitudinal traits remains a major obstacle.

Longitudinal traits (ie, time-series phenotypes) provide valuable information regarding the progression of diseases. Traditionally, such longitudinal data can be analyzed using the so-called cross-sectional strategies. In particular, such methods involve repeating the same analysis at various, specific points in time. Since at each time point the trait under consideration reduces to a scalar, methods such as inverse-probability clustering can be conducted for association screening. Then, variants can be selected based on the results from each time point. The assumption underlying this type of strategy is that genetic variants maintain similar influences at different time points. However, it is more likely that those variants influence the pattern of the traits across time; for example, a group of variants may affect how blood pressure changes in a time-dependent manner. Cross-sectional analysis may fail under such circumstances. A method that takes full consideration of the longitudinal nature of traits is thus desired to capture such genetics-time interactions.

In this paper, we propose a dual-clustering framework, which highlights both rare variants and the longitudinal structure of traits. By "dual" clustering, we mean individuals are clustered based on both genotypic information through inverse-probability weighting and longitudinal traits through ordinary hierarchical clustering. Association between the 2 sets of partition labels can then be readily evaluated using existing single-marker and scalar-trait association methods, such as one-way analysis of variance (ANOVA) or the partition retention (PR) method [5,6]. We apply the proposed approach to the simulated data of the Genetic Analysis Workshop 18 (GAW18) and compare the obtained results with those obtained by SKAT. The comparison produces some interesting findings.

## Methods

### Data set

The simulated data set of GAW18 is a combination of real WGS data and simulated longitudinal traits. The sequence data is drawn from T2D-GENES Project 2. In this paper, we use the dosage genotype data on chromosome 3, which include 773,088 single-nucleotide polymorphisms (SNPs) that can be mapped to the genome. Two hundred phenotype sets were simulated based on genotype data. For each simulated data set, we analyze systolic blood pressure (SBP) and diastolic blood pressure (DBP), each with measurements at 3 time points, for 849 related subjects. We map the SNPs to its host gene, resulting in 1426 genes.

### Inverse-probability clustering based on genotypes

Let *g_ik _*= 0, 1, or 2 represent the number of minor alleles at SNP *k *for individual *i*, and let *p_k _* be the observed MAF of SNP *k*. We define the inverse-probability weighted similarity score between individuals *i *and *j *based on SNP *k *as:

(1) $$\text{sim}\left(i,j;k\right)=\left\{\begin{array}{c} \frac{2}{p_{k}^{2}},\text{ if }g_{ik}=g_{jk}=0\\ \frac{1}{2}\left[\frac{1}{p_{k}^{2}}+\frac{1}{{\left(1-p_{k}\right)}^{2}}-\frac{1}{p_{k}\left(1-p_{k}\right)}\right],\text{ if }g_{ik}=g_{jk}=1\\ \frac{2}{{\left(1-p_{k}\right)}^{2}},\text{ if }g_{ik}=g_{jk}=2\\ \frac{1}{{\left(1-p_{k}\right)}^{2}}-\frac{1}{2p_{k}\left(1-p_{k}\right)},\text{ if }g_{ik}+g_{jk}=1\\ -\frac{1}{p_{k}\left(1-p_{k}\right)},\text{ if }g_{ik}=0,g_{jk}=2\text{ or }g_{ik}=2,g_{jk}=0\\ \frac{1}{p_{k}^{2}}-\frac{1}{2p_{k}\left(1-p_{k}\right)},\text{ if }g_{ik}+g_{jk}=3 \end{array}\right)$$

**Figure 1:**
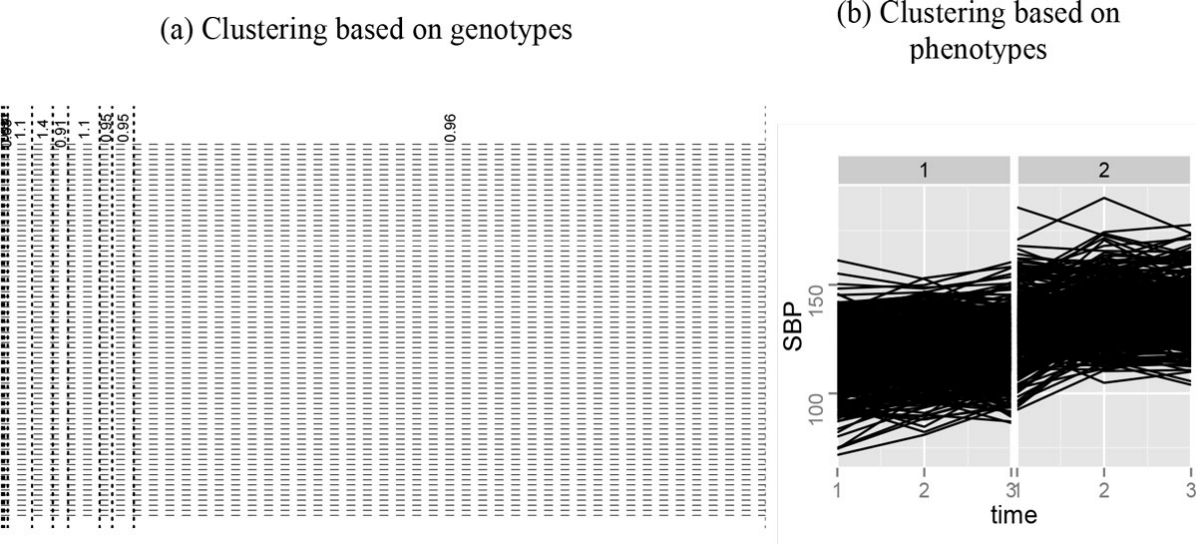
**Clustering of individuals using SNPs with MAFs between 0.01 and 0.05 for MAP4. **A, Shown are 10 clusters, with the numbers at the top odds ratios within each partition block based on blood pressures. Each row is a SNP, and each column is an individual. SNPs are ordered with decreasing MAFs (from top to bottom). Green vertical bars indicate subjects with higher blood pressures (see text). Genotype *aa *is plotted in red, *aA *is plotted in blue, and *AA *is plotted in white (*a *denotes the minor allele). The partitions of the 849 individuals are indicated by dotted lines. Most partition elements are driven by similarity on rarer SNPs but not on more common SNPs. B, Clustering of individuals using their SBP curves from the first simulation. It can be seen that individuals are reasonably grouped into 1 high blood pressure cluster and 1 low blood pressure cluster.

The definition in equation (1) highlights the influence of rare variants, and the genotypic similarity on minor alleles, but not that on major alleles [4]. For a given gene *G*, the similarity between individuals *i *and *j *is defined as the sum of the similarity scores on SNPs within that gene: $\text{sim}\left(i,j\right)=\sum_{k\in G}\text{sim}\left(i,j;k\right)$. For the 849 individuals, pairwise similarity scores, sim(*i, j*)'s, are evaluated first and then converted to a distance measure using the transformation $d\left(i,j\right)=-\text{sim}\left(i,j\right)+max\left(\text{sim}\left(m,n\right)\right)$, such that the pair with the largest similarity has distance 0. Other bounded monotone-decreasing transformations can also be applied, such as exponential transformations adopted in our previous work [4]. We then conduct hierarchical clustering based on the above distances using Ward's method [7], and partition individuals into groups by cutting the hierarchical clustering tree into a prespecified number of groups (we consider partition sizes of 5 to 10). Figure 1A providesan example using MAP4.

### Hierarchical clustering based on longitudinal phenotypes

The main difficulty of dealing with longitudinal traits is that most existing association methods only consider scalar phenotypes. Thus it is natural to transform longitudinal traits into some 1-dimensional summary statistics. Here we adopt ordinary hierarchical clustering using phenotype vectors and treat the resulting class labels as a summary statistic. Because hierarchical clustering uses the whole longitudinal trait as features, we expect that it can capture the structure contained in the phenotypes. In this study, we cluster the 849 individuals into 2 groups. Results show that these 2 groups can be treated as with high and low blood pressures (see Figure 1B). Our main focus here is a strategy that turns longitudinal traits into 1-dimensional summaries. Other dimension-reduction techniques can also be used for this task. We choose to adopt hierarchical clustering for illustration purpose here because of its simplicity, and we get reasonable results (see **Results**).

### Association analysis based on obtained clusters

After clustering individuals based on both genotype and phenotype, for each gene we test the association between the corresponding 2 sets of partition indices. We consider one-way ANOVA and the partition-retention method [5,6]. The partition-retention method is based on an association measure *I* being defined as between an outcome variable *Y *and a partition Π. Specifically, $I=\sum_{\Pi_{i}}\frac{n_{i}}{n}\frac{{\left({Ȳ}_{i}-Ȳ\right)}^{2}}{s^{2}/n_{i}}$, where *n_i _*is the number of individuals in partition element *i*, and $\bar{Y_{i}}$ is the sample mean of element *i*. *Ȳ *and *s* are the sample mean and standard deviation of all *n *individuals, respectively. Here we take the variable that indicates which cluster an individual is in from longitudinal traits as *Y*. Intuitively, PR's *I* as defined above evaluates the amount of influence a particular gene has on the longitudinal trait indexed by *Y*.

### Sequence kernel association test

We also analyze the data using the linear SKAT [3] for comparison purpose. We briefly describe this method here. Following the same notation, a similarity score between individuals *i *and *j *based on SNP *k *can be defined as: $\text{sim}\left(i,j;k\right)=w_{k}g_{i}g_{j}$, where *w_k _*is a weight for the *k*^th^SNP. The weights (*w_k_*s) are defined based on the corresponding MAFs, such that the influence of rare variants can be boosted, an idea morally similar to the similarity scores defined in equation (1). For a particular gene, similarity between 2 individuals can be defined by the same summation as in our method.

SKAT uses the variance-component score statistic based on the above similarity scores to test for association between genotypic variants and a scalar trait. We treat the cluster indices from the longitudinal traits as the response variable in order to apply SKAT to GAW18 data. More details on SKAT can be found in Ref. [3].

## Results

We first apply the proposed method to the WGS dosage data including all the 773,088 SNPs and the SBP trait. Three genes are discovered after Bonferroni correction, of which 1 gene, Y_RNA, is significant in 15 of the 200 replicates. It turns out that this gene resides within MAP4, which has the strongest signal in the simulated model, and produces a noncoding RNA.

**Figure 2:**
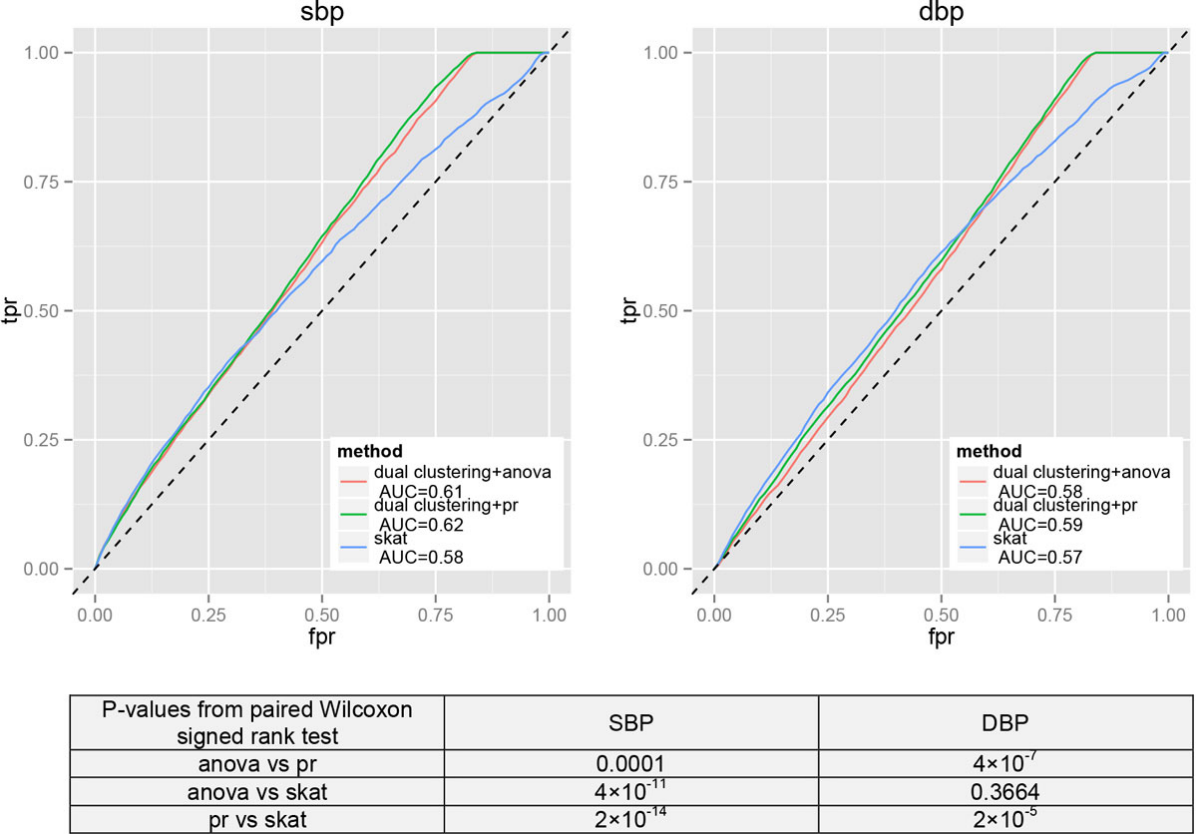
**Average ROC curves across simulation replicates for 3 methods**. Shown are results by 10 clusters using inverse-probability weighting. Areas under the curve (AUCs) by different methods are compared using paired Wilcoxon signed rank tests based on the 200 replicates, with the resulting *p* values shown in the table below. *fpr*, ie, false positive rate, is the ratio of the number of claimed causal genes and the number of true noncausal genes; *tpr*, ie, true positive rate, is the ratio of the number of claimed causal genes and the number of true causal genes.

One reason for the relatively few significant genes obtained above may be that there is a very high density of variants within most genes. We then conduct similar analysis using only SNPs with MAFs between 0.01 and 0.05 to increase power. SBP and DBP are regressed on age, sex, age × sex, and medication, and the residuals are used in the clustering analysis. For method comparison, we treat genes containing at least 1 causal SNP in the simulated model as causal genes, resulting in 21 genes for SBP and 26 genes for DBP. We compare the receiver operating characteristic (ROC) curves by the proposed dual-clustering framework and SKAT (Figure 2). SKAT cannot get results for some of the replicates. It can be seen from Figure 2 that all the 3 methods have relatively low power, among which our dual-clustering approach with PR's *I *has a bigger area under curve (AUC). Results are similar for other partition sizes resulted from inverse-probability clustering.

## Discussion

We propose a dual-clustering framework for gene-based association analysis with WGS and longitudinal traits. The first clustering is based on the inverse-probability weighted similarities, which automatically increase weights for rare variants. The similarity scores are calculated from empirical MAF estimates. If better estimates are available, the proposed method can incorporate the better estimates to achieve improved power. The second clustering treats trait vectors of individuals as features, which accounts for the longitudinal nature of the phenotypes. Individuals are then partitioned based on their genetic similarity on the SNPs in a gene, as well as the similarity of their traits. These 2 partitions are then used to calculate association between a gene and a longitudinal trait.

Our proposed framework is actually quite general. We define the similarity measure based on inverse-probability weighting. Other similarity measures, such as the one used in SKAT, can also be incorporated into our framework. Other distance-based clustering approaches can be adopted for the first clustering based on similarity measures. The proposed similarities can detect variants with variable directions of the effects. For longitudinal traits, we choose hierarchical clustering because of its simplicity. Hierarchical clustering does not take into account the correlation induced by time. Considering there are only 3 time points in the GAW18 data, we believe that not much information has been lost. If more time points are available, time-series clustering methods can be used (see Ref. [7] for a survey on commonly used time-series clustering algorithms). More generally, we use clustering as a means of summarization, so other summarization strategies can also be integrated into the proposed framework. After obtaining the 2 sets of clustering indices, any association method can be used to measure the association between them. In this paper, we choose ANOVA and PR's *I*. The obtained results are similar but a little better than that from SKAT in terms of ROC curves (see Figure 2). SKAT shows superiority to more traditional methods in the simulation studies presented in Ref. [3]. Many of those traditional methods assume that causal variants have effects with the same direction and magnitude, and do not consider the potential effects of rarer variants to boost power. The purpose of the current study is not to show the absolute superiority of our method, but rather to present a general framework that can incorporate different choices of similarities and association measures, such as that from SKAT.

Although the simulation model did not take family structures into account, the ANOVA *p *values may be inflated as a consequence of such structures. However, *p *values will be inflated (if any) for both causal and noncausal variants. Therefore, the main conclusion based on ROC curves is still valid. In practice, we suggest evaluating *p *values using permutations and controlling the false discovery rate in order to have better sensitivity to real genetic signals. This may introduce more computational burden, but it is worth mentioning that the 2 clustering tasks can be done independently and simultaneously so that the computational time can be reduced. Multilevel models with Markov chain Monte Carlo (MCMC) techniques may also address the multiple comparisons problem encountered here by partial pooling [8].

## Conclusions

The methods we experimented on have relatively low power on this particular data set. Our framework obtains slightly better results in terms of AUC. It is worth applying the proposed methods to other data sets for a comprehensive understanding of its performance.

## Competing interests

The authors declare that they have no competing interests.

## Authors' contributions

TZ and YL conceived of and designed the study, performed the statistical analysis, and drafted the manuscript. TZ participated in its coordination. TZ, YL, CHH, SHL and IH discussed the results. All authors read and approved the final manuscript.
