# Correction: A dual clustering framework for association screening with whole genome sequencing data and longitudinal traits

**DOI:** 10.1186/1753-6561-8-S1-S112

**Published:** 2014-07-24

**Authors:** Ying Liu, Chien Hsun Huang, Inchi Hu, Shaw-Hwa Lo, Tian Zheng

**Affiliations:** 1 Department of Statistics, Columbia University, New York, NY 10027, USA; 2 ISOM, Hong Kong University of Science and Technology, Kowloon, Hong Kong

## Correction

For the previous publication of our article [1], Figure 1 was incorrectly processed as grayscale. We present, here in this correction, the original Figure in full color.

**Figure 1:**
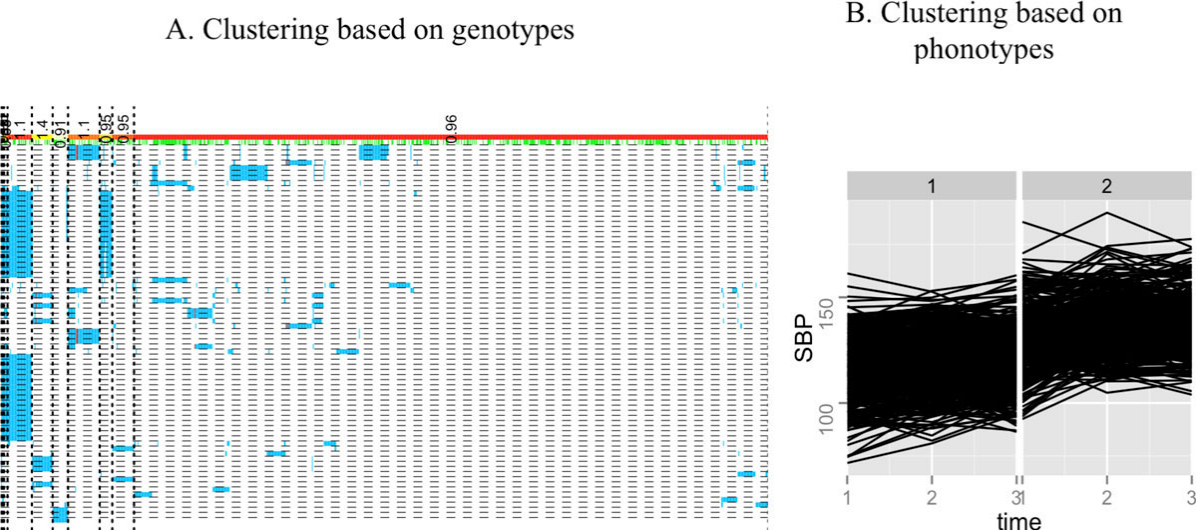
**Clustering of individuals using SNPs with MAFs between 0.01 and 0.05 for MAP4**. A, Shown are 10 clusters, with the numbers at the top odds ratios within each partition block based on blood pressures. Each row is a SNP, and each column is an individual. SNPs are ordered with decreasing MAFs (from top to bottom). Green vertical bars indicate subjects with higher blood pressures (see text). Genotype *aa *is plotted in red, *aA *is plotted in blue, and *AA *is plotted in white (*a *denotes the minor allele). The partitions of the 849 individuals are indicated by dotted lines. Most partition elements are driven by similarity on rarer SNPs but not on more common SNPs. B, Clustering of individuals using their SBP curves from the first simulation. It can be seen that individuals are reasonably grouped into 1 high blood pressure cluster and 1 low blood pressure cluster.

## Notes

This article has been published as part of *BMC Proceedings *Volume 8 Supplement 1, 2014: Genetic Analysis Workshop 18. The full contents of the supplement are available online at http://www.biomedcentral.com/bmcproc/supplements/8/S1.
